# Treatment outcomes among adults with HIV/non-communicable disease multimorbidity attending integrated care clubs in Cape Town, South Africa

**DOI:** 10.1186/s12981-021-00387-3

**Published:** 2021-10-14

**Authors:** Blessings Gausi, Natacha Berkowitz, Nisha Jacob, Tolu Oni

**Affiliations:** 1 School of Public Health and Family Medicine, Faculty of Health Sciences, University of Cape Town, Anzio Road, Observatory 7925, Cape Town, South Africa; 2 MRC Epidemiology Unit, University of Cambridge, Cambridge, UK

**Keywords:** HIV/AIDS, Hypertension, Diabetes mellitus, Non-communicable diseases, Multimorbidity, Integrated care, Adherence clubs, South Africa

## Abstract

**Background:**

The growing burden of the HIV and non-communicable disease (NCD) syndemic in Sub- Saharan Africa has necessitated introduction of integrated models of care in order to leverage existing HIV care infrastructure for NCDs. However, there is paucity of literature on treatment outcomes for multimorbid patients attending integrated care. We describe 12-month treatment outcomes among multimorbid patients attending integrated antiretroviral treatment (ART) and NCD clubs in Cape Town, South Africa.

**Methods:**

As part of an integrated clubs (IC) model pilot implemented in 2016 by the local government at two primary health care clinics in Cape Town, we identified all multimorbid patients who were enrolled for IC for at least 12 months by August 2017. Mean adherence percentages (using proxy of medication collection and attendance of club visits) and optimal disease control (defined as the proportion of participants achieving optimal blood pressure, glycosylated haemoglobin control and HIV viral load suppression where appropriate) were calculated at 12 months before, at the point of IC enrolment and 12 months after IC enrolment. Predictors of NCD control 12 months post IC enrolment were investigated using multivariable logistic regression.

**Results:**

As of 31 August 2017, 247 HIV-infected patients in total had been enrolled into IC for at least 12 months. Of these, 221 (89.5%) had hypertension, 4 (1.6%) had diabetes mellitus and 22 (8.9%) had both diseases. Adherence was maintained before and after IC enrolment with mean adherence percentages of 92.2% and 94.2% respectively. HIV viral suppression rates were 98.6%, 99.5% and 99.4% at the three time points respectively. Retention in care was high with 6.9% lost to follow up at 12 months post IC enrolment. Across the 3 time-points, optimal blood pressure control was achieved in 43.1%, 58.9% and 49.4% of participants while optimal glycaemic control was achieved in 47.4%, 87.5% and 53.3% of participants with diabetes respectively. Multivariable logistic analyses showed no independent variables significantly associated with NCD control.

**Conclusion:**

Multimorbid adults living with HIV achieved high levels of HIV control in integrated HIV and NCD clubs. However, intensified interventions are needed to maintain NCD control in the long term.

**Supplementary Information:**

The online version contains supplementary material available at 10.1186/s12981-021-00387-3.

## Background

South Africa has the world’s largest burden of HIV, with an HIV prevalence of 12% [1]. Consequently, it has the largest antiretroviral treatment (ART) program in the world [2], with 3.4 million patients receiving ART care at no cost to the individual [3]. Since the advent of effective ART, HIV has become a chronic, manageable illness, with lifespan approaching that of HIV-negative persons [4]. This increased lifespan, along with the aging effect of HIV and drug interactions [5, 6], has resulted in an increased risk of developing non-communicable diseases (NCDs) and consequentially an increased burden of multimorbidity (MM) among people living with HIV (PLHIV). Multimorbidity is defined as the co-existence of more than one chronic condition in one person [7].

Previous research has shown that there is a significant burden of MM in South Africa with prevalence estimates ranging from 22.6 to 48.4% [8, 9]. A study investigating MM in Khayelitsha, Western Cape (the setting of this study), found high prevalence of comorbid hypertension and diabetes (19.7% and 12.3% respectively) amongst PLHIV on ART aged between 18–35 [9]. This dual epidemic of communicable and NCDs has knock-on effects on the current health system which is ill-equipped to cope with the inherent complexity of MM [10]. The high comorbidity among PLHIV highlights the need to integrate care of these conditions into routine ART management. In the Western Cape, South Africa, PLHIV without NCD diagnoses attend regular ART medical adherence clubs (MACs) which comprise 25–30 PLHIV who have been on ART for at least 6 months with suppressed viral loads (VL). MACs involve both task-shifting and decentralization of care at the primary health care level and have been shown to decongest healthcare facilities [11], improve retention in care [12], maintain virologic suppression [13], be cost effective [14] and acceptable to both patients and health care workers [15].

In response to the rising burden of MM, some clinics in the city of Cape Town have sought to achieve integration; piloting a novel model of care that adapts the MAC model to integrate HIV and NCD care. The structure and eligibility for HIV/NCD integrated care (IC) is similar to the MAC model with the eligibility criteria of a diagnosis of NCD such as Diabetes Mellitus (DM) or hypertension (HTN) or both (DMHTN) in patients with HIV Prior to this pilot, the standard of care for a MAC attendee with comorbid NCD comprised attending a MAC club for ART care and a different outpatient appointment for NCD care often on a different day and sometimes, at a different health facility. In order to minimize logistical barriers to accessing NCD care for multimorbid patients and improve efficiency of health care delivery, PLHIV and clinically controlled NCD were enrolled in new IC clubs that provide HIV and NCD care concurrently. Initially, in the piloting phase, IC club participants were recruited from ART only adherence clubs. Upon successful implementation of the IC club pilots, eligibility was expanding to include any patient with clinically controlled HIV and NCD diagnoses, irrespective of whether they were part of ART only adherence clubs. Majority (80.2%) were initially attending ART only adherence clubs whereas 19.8% were enrolled from standard of care.

We have  comprehensively described various models of integrated HIV/NCD care that exist to date  in our scoping review [Duration with NCD [44]]. The models of care for HIV/NCD integration can be summarized into four as: (i) integration of NCD screening and treatment services into established HIV centres [16–18]; (ii) integration of HIV screening and treatment services into established NCD centres; (iii) simultaneous integration of HIV and NCD services at health facilities [19–21] and (iv) integrated HIV and NCD care specifically for multi-morbid patients [22, 23]. Noteworthy is the fact that only two studies worldwide have reported treatment outcomes among PLHIV with comorbid DM or HTN (model iv) [22, 23], and no study has evaluated treatment outcomes among PLHIV with comorbid NCDs attending IC in particular.

It is against this background that we sought to investigate the long term patient outcomes, among PLHIV with MM attending IC model of care since implementation in Cape Town. These long-term outcomes include, but are not limited to, medication adherence, retention in care, loss to follow up, HIV viral suppression and NCD control. Thus, it is not known if IC improves or at least maintains the desired clinical HIV and NCD outcomes compared to standard of care (MAC plus separate NCD care) in multimorbid PLHIV. Such evidence is needed to inform action by health program managers and policy makers in order to adopt, implement and scale up IC for PLHIV with NCD multimorbidity, particularly in high HIV-burden settings undergoing rapid epidemiological transition. In this study, we addressed this knowledge gap by assessing clinical outcomes in patients with HIV and comorbid DM and/or HTN before and after 12 months of receiving the IC model of care in Cape Town, South Africa.

## Methods

### Study setting and design

We conducted an observational retrospective cohort study that followed up patients living with HIV and comorbid NCD before and upon enrolment into integrated clubs(IC). Routine clinic data were collected from patient medical records and club registers in order to assess clinical outcomes for PLHIV 12 months before, at the point of IC entry, and 12 months after attending IC at two City of Cape Town primary care clinics(clinic A and B, names withheld to maintain participant confidentiality) in Cape Town, South Africa. The two public health facilities are under the governance of the City of Cape Town Health Department and both ART and NCD treatment are provided for free. They are based in the peri-urban township of Khayelitsha which has a population of approximately 500,000 [24] and an estimated antenatal HIV prevalence of 34.3% [25]. The study was approved by the University of Cape Town, Faculty of Health Sciences Human Research Ethics committee (HREC Ref no: 497/2019).

### Sampling and statistical power

Study participants were all adults over 18 years old, who had documented HIV-infection and a diagnosis of either DM or HTN or both, had attended MACs for HIV care or had been on ART for at least 6 months before they were enrolled into IC and had been enrolled in ICs for at least 12 months as of August 2017. All adult patients who attended IC at the two pilot sites and met these inclusion criteria were included in the study. As there were a limited number of patients with multimorbidity attending the study clinics and no published studies that have investigated patient outcomes among comorbid PLHIV attending IC, we approached the entire population of adult patients who attended IC at the two pilot sites for consent and recruitment into the study.

### Data collection

Patients with comorbid diagnosis of HTN, DM or both (DMHTN) were identified from the IC club registers and clinic folders. Diagnosis of HTN or DM was made by facility clinicians based on the South African Primary Care (PACK) guidelines [27]. Outcome measures (blood pressure (BP), VL, and glycosylated heamoglobin (HbA1c)) were extracted from electronic and paper clinic records from September 2016 to August 2017. Participant data were collected retrospectively from participants clinical records and club registers. Clinical procedures routinely undertaken in MAC and IC clubs are illustrated in Table 1. Anonymized data were captured by a trained study team onto a Redcap electronic database hosted at the University of Cape Town [28].

**Table 1:** Adherence club procedures

	ART medical adherence club (MAC)		Integrated HIV/NCD Club (IC)
Club formation	Approximately 25 patients are recruited into a club simultaneously and initiated into the club process together		
Club admission criteria	HIV-positive at least 6 months on ART with suppressed viral load (VL)		HIV-positive with DM and/or HTN, at least 6 months on ART with suppressed VL. Blood pressure (BP) < 140/90 mmHg and HbA1c < 9%
Number of Club visits per year	Total = 5 3 medication collections 1 medication collection and clinical examination 1 medication collection and phlebotomy		
Bloods tests conducted at phlebotomy visit	Viral Load and safety bloods (Liver function, renal function and full blood count depending on ART regimen)^a^		Viral Load and safety bloods (Liver function, renal function and full blood count depending on ART regimen) HbA1c^b^ Creatinine^b^
Procedures at each clinical visit	Weight, BP, screening for tuberculosis symptoms and ART side effects, HIV health education and adherence counselling at each visit by lay-counsellor^c^		
Staff providing care	Lay-counsellor for medication collections Professional Nurse practitioner for clinical examination and phlebotomy Medical officer to review complicated patients		

*HbA1c* glycosylated heamoglobin

^a^Based on provincial ART guidelines [26]

^b^Based on Primary Care “PACK” guidelines [27]

^c^No specific NCD counselling for multimorbid patients provided

Patient demographic (age, sex), anthropometric (weight, height), disease-related (NCD diagnoses, time since NCD diagnosis, World Health Organization (WHO) stage at HIV diagnosis, CD4 count at HIV diagnosis, time since HIV diagnosis, duration on ART), and IC club-related (IC club registration date, IC clinic) variables were extracted. Adherence was assessed 12 months before and 12 months after IC enrolment. Prior to IC enrolment, we used medication collection as a proxy for adherence. After IC enrolment, adherence to club visits (club attendance), extracted from club registers was used as a proxy for adherence. Good adherence was defined as proportion of attended visits of > 80%, according to WHO classification of adherence to long term therapy [29].

Variables related to disease control were measured at 12 months before IC enrolment, at IC enrolment and at 12 months after IC enrolment. HIV control was defined as having a viral load of < 1000 copies/ml [26] whereas NCD control was defined by the Society of Endocrinology, Metabolism and Diabetes of South Africa (SEMSA) targets for BP (BP < 140/90 mmHg) and HbA1c (HbA1c < 7.5%) among persons with hypertension and diabetes respectively [30].

### Statistical analysis

Given small numbers, participant data from both clinics were pooled for analysis. Categorical variables were described using frequencies and proportions, normally distributed continuous variables using means and 95 percent confidence intervals (95% CI), and non-parametric continuous variables using medians and interquartile ranges (IQR). Adherence to scheduled appointments was calculated as the proportion of appointments attended by each participant from 12 months prior to IC enrolment up to the point of IC enrolment, and from IC enrolment to 12 months later. Mean adherence percentages were calculated for the 12 months prior to IC enrolment and 12 months post IC enrolment and compared using a paired student t-test. The proportion of participants with optimally controlled NCDs according to SEMSA targets was calculated cross-sectionally for the three time points: (i) at 12 months before IC enrolment, (ii) at IC enrolment and (iii) at 12 months post IC enrolment.

The proportions of participants with optimally controlled NCD at IC enrolment and at 12 months post IC enrolment were compared using the Chi-squared test under the null hypothesis that IC enrolment maintains clinical control of multimorbidity. Chi-square or Fisher’s exact tests were used where appropriate to explore baseline variables associated with control of NCD at 12 months post IC. Univariate logistic regression was used to explore factors associated with comorbidity control and crude odds ratios calculated to identify independent variables that yielded a p-value of ≤ 0.2. These variables were used to build a multivariate logistic model to estimate adjusted odds ratios and 95% CI for predictors of clinical control of NCD, with the outcome variable (NCD control) categorised as 1 if HTN or DM or both, were optimally controlled at 12 months post IC enrolment and as 0 if otherwise. Significance testing was performed using 2-sided p-values at α of 0.05. All statistical analyses were conducted in STATA 15.0 (Stata Corp LP, College Station, TX).

## Results

As of 31 August 2017, a total of 247 patients had been enrolled into IC clubs for at least 12 months at the two primary health care facilities (Table 2). There were no significant differences in demographic and clinical characteristics between patients at the two facilities at baseline, with the exception of duration with NCD (patients receiving care at Clinic B had relatively more recent NCD diagnoses compared to patients at Clinic A) (Table 2).

**Table 2:** Demographic and clinical characteristics of study participants at baseline

Variable^a^	Clinic A (n = 71)	Clinic B (n = 176)	Total (N = 247)	p-value
Age (years), mean ± SD	48.4 ± 8.4	45.9 ± 8.7	46.7 ± 8.6	0.051
Sex, male	18 (25.4)	41 (23.3)	59 (23.9)	0.865
Comorbidity				
DM only	0 (0)	4 (2.3)	4 (1.6)	
HTN only	63 (88.7)	158 (89.8)	221 (89.5)	
DM and HTN	8 (11.3)	14 (7.9)	22 (8.9)	0.326
Time with NCD (years)				
0–5	43 (60.6)	124 (70.5)	167 (67.6)	
6–10	20 (28.2)	4 (2.3)	24 (9.7)	
> 10	3 (4.2)	3 (1.7)	6 (2.4)	** < 0.001**
Missing	5 (7.0)	45 (25.6)	50 (20.2)	
Time with HIV (years)				
0–5	31 (43.7)	91 (51.7)	122 (49.4)	
6–10	28 (39.4)	49 (27.8)	77 (31.7)	
> 10	11 (15.5)	15 (8.5)	26 (10.5)	
Missing	1 (1.4)	21 (11.9)	22 (8.9)	0.113
Time on ART (years)				
0–5	48 (67.6)	139 (78.9)	187 (75.7)	
6–10	22 (30.9)	36 (20.5)	58 (23.5)	
> 10	1 (1.1)	1 (0.6)	2 (0.8)	0.155
WHO stage at HIV diagnosis				
0	14 (19.7)	60 (34.1)	74 (29.9)	
1	21 (29.6)	53 (30.1)	74 (29.9)	
2	28 (39.5)	43 (24.4)	71 (28.7)	
3	6 (8.5)	15 (8.5)	21 (8.5)	
4	2 (2.8)	5 (2.8)	7 (2.8)	0.103
CD4 count at HIV diagnosis^b^				
< 350	40 (56.3)	89 (50.6)	129 (52.2)	
≥ 350	25 (35.2)	42 (23.9)	67 (27.1)	
Missing	6 (8.5)	45 (25.6)	51 (20.7)	0.374
Median CD4 count at HIV diagnosis (IQR)^b^	294 (121–419)	249 (150–393)	261.5 (146–403.5)	
HIV suppression rates	64 (90.1)	147 (83.5)	211 (85.4)	
Missing	7 (9.9)	28 (15.9)	35 (14.2)	0.627

*HTN* Hypertension, *DM* Diabetes mellitus type 2, *DMHTN* dual diagnosis of HTN and Diabetes Mellitus type 2, *NCD* non-communicable disease which implies either DM or HTN or both in this case, *SD* standard deviation of the mean

^a^Characteristics are described as n (%) where n is number of participants with the characteristic and % is percentage of the study population with the given characteristic

^b^Cells/µL

Bold p values indicated statitistical significance

### Patterns of multi-morbidity and treatment

Of the 247 patients, 221 (89.5%) had comorbid HTN, and 22 (8.9%) had a triple burden of HIV, DM and HTN. Four (1.6%) patients had DM only. The median time with multimorbidity regardless of type of NCD, was 3 (IQR (2–4) years among patients with available data on duration since NCD diagnosis (n = 197) (Table 2).

Among those with HTN, 95.5% received pharmacological therapy. The majority of patients were being treated with Hydrochlorothiazide alone (82.9%), Hydrochlorothiazide and Enalapril (9.9%) whereas (6.6%) were treated with a combination of Hydrochlorothiazide, Enalapril and Amlodipine. All patients with comorbid DM were treated with oral anti-glycaemic agents. Pharmacological therapy for HTN among patients with HTN and DM was similar to that among patients with HTN only (Additional file 1: Table S1).

### Outcome variable measurements

**Fig. 1:**
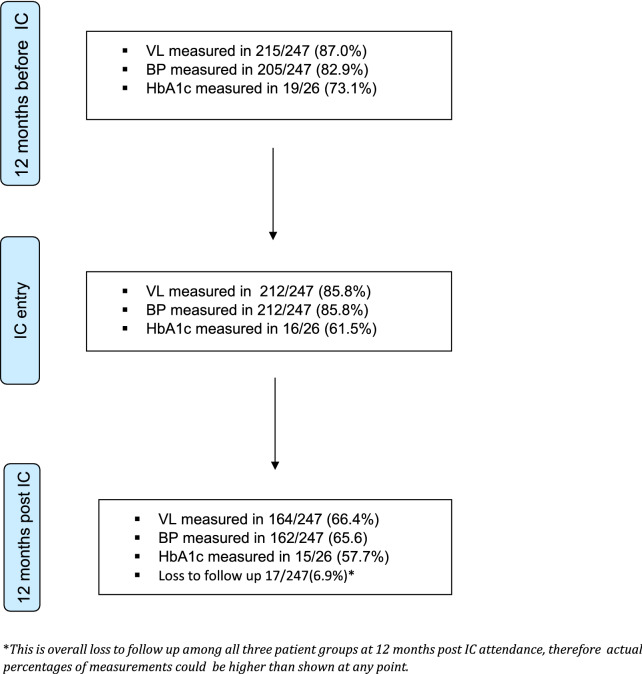
Available records for viral loads (VL), blood pressure (BP) and glycosylated heamoglobin (HbA1c) at three time points in the study. HbA1c measured in patients with DM

Documented measurements for our outcome variables (viral load, blood pressure and HbA1c) varied at 12 months prior to IC, IC entry and 12 months post IC. Figure 1 shows number and percentage of participants with documented outcome variable measurements at the three end points. At the end of 12 months post IC, 17 (6.9%) participants overall, were lost to follow up.

### Adherence to medication

As adherence proportions before and after IC were normally distributed, a paired t-test was used to assess differences in mean adherence. Mean adherence proportions before and after IC enrolment were 92.2% and 94.2% respectively. There was no significant difference in mean adherence proportions before and after attending IC (p = 0.223) either overall or by patterns of MM.

We compared the proportion of patients with good adherence (adherence > 80%) before and after attending IC and found no difference in the percentage of patients with good adherence (91.9% and 90.3% respectively, p = 0.693).This observation did also not change when categorised by patterns of MM.

### HIV control

In our study population, 215/247 (87.0%) had documented viral load testing at 12 months prior to IC enrolment. Of these, 212 (98.6%) were virally suppressed. Similarly, 212 (85.8%) patients had documented viral load testing at IC enrolment and 211 (99.5%) were virally suppressed. Twelve months after IC, 164 (66.4) had documented viral load testing of which 163 (99.4%) were virally suppressed. Of the participants enrolled in IC, 93.1% were retained in care at 1-year post IC enrolment with 6.9% lost to follow up (Fig. 1). HIV control did not differ by patterns of MM.

### Blood pressure control

**Fig. 2:**
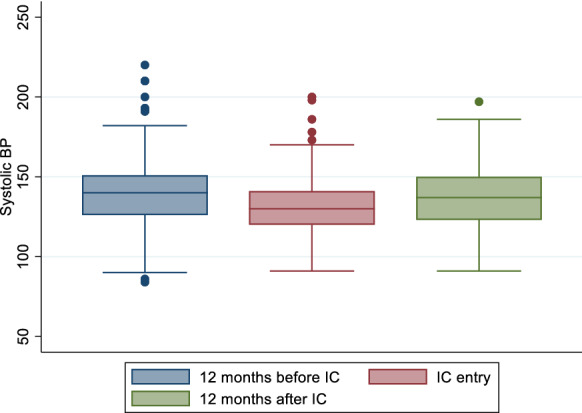
Mean systolic blood pressure at three time points: 12 months before, at entry and 12 months after entry to integrated club

**Fig. 3:**
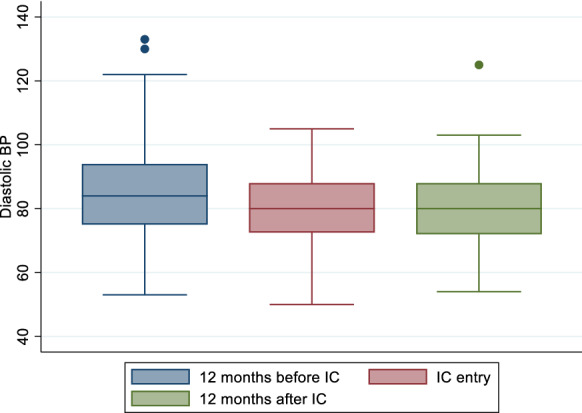
Mean diastolic blood pressure at three time points: 12 months before, at entry and 12 months after entry to integrated club

Blood pressure control was assessed among all participants including those with only DM as this is part of routine clinical evaluation for comprehensive diabetes care. Of the 247 participants, 205 (82.9%), 212 (85.8%) and 162 (65.6%) had blood pressure measurements at 1-year before IC, at IC enrolment and 1-year post IC enrolment respectively (Fig. 1). Among those with blood pressure measurement, 43.4%, had controlled BP at 1 year before IC, 58.9% had controlled BP at IC entry while 49.4% had controlled their BP at 1 year post IC enrolment. Mean systolic blood pressure (SBP) was 139.9 mmHg [95% CI (136.9–142.9 mmHg)], 132.1 mmHg (95% CI (129.7–134.6 mmHg)) and 136.7 mmHg [95% CI (133.7–139.6 mmHg)] at these time points respectively; demonstrating a pattern of decrease up to the point of enrolment and increasing 1 year later. This pattern was also observed for diastolic blood pressure (DBP) with the mean DBP of 84.7 mmHg [95% CI (82.7–86.6 mmHg)], 79.7 mmHg [95% CI (78.2–81.2 mmHg)] and 80.1 mmHg [95% CI (78.4–81.9 mmHg)] at 1-year before IC, at IC enrolment and 1-year post IC enrolment respectively (Figs. 2 and 3).

The pattern of increasing SBP at 1-year post-IC enrolment compared to IC entry was statistically significant (p = 0.009) whereas the pattern of increasing DBP 1-year post-IC enrolment compared to IC entry was not statistically significant (p = 0.370).

**Fig. 4:**
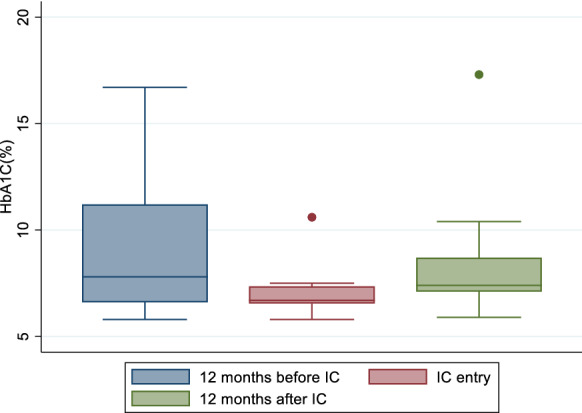
Mean glycosylated heamoglobin (HbA1c) at three time points: 12 months before, at entry and 12 months after entry to integrated club

BP control declined by 9.5% at 1-year post IC (p = 0.033) with 43.4% [95% CI (34.6–50.5%)], 58.9% [95% CI (52.0–65.7%)] and 49.4% [95% CI (41.5–57.3%)] of participants with optimally controlled BP at 1-year before IC enrolment, at IC enrolment and at 1-year post IC enrolment respectively (Fig. 4).

Comparing optimal BP control at 1-year post IC enrolment in participants with HTN only with those with DM and HTN, there was no significant difference in BP control among the two patient groups (64.3% vs 47.6% respectively, p = 0.117).

### Diabetes control

**Fig. 5:**
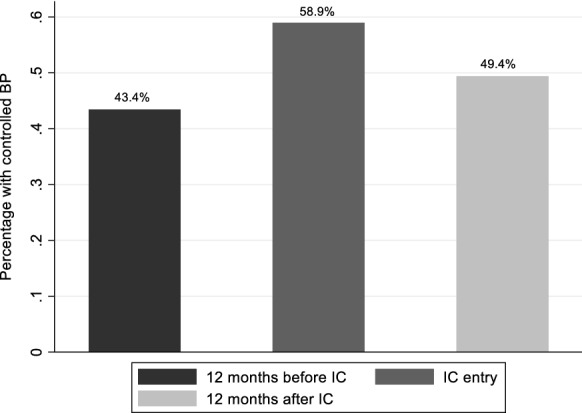
Proportion of participants with controlled Blood Pressure (BP) at three time points

Of 26 patients with DM (either DM alone or DMHTN), 19 (73.1%), 16 (61.5%) and 15 (57.7%) had recorded HbA1c measurement 12 months prior to IC enrolment, at IC enrolment and at 1-year post IC enrolment respectively. Mean HbA1c among all patients with DM (either DM or DMHTN) was 8.9% [95% CI (7.3–10.5%)], 7.0% [95% CI (6.4–7.6%)], and 8.3% [95% CI (6.8–9.9%)] at these time points (Fig. 5). The proportion of patients with optimally controlled DM at 1-year post IC enrolment was significantly lower compared to the same proportion at IC enrolment (p = 0.018) with more than 30% of participants who had initially achieved optimal glycaemic control at IC enrolment having sub-optimal glycaemic control 1-year following IC enrolment [47.4% (95% CI (24.5–71.1%)], 87.5% [95% CI (61.7–98.5)] and 53.3% (95% CI (26.6–78.7) at 1-year pre IC enrolment, at IC enrolment and at 1-year post IC enrolment respectively).

### Factors associated with optimal NCD control

Three variables at baseline were significantly associated with control of NCD after 12 months of attending IC. These are WHO stage at baseline, time with NCD and facility (Table 3). Clinic A was associated with better NCD control compared to clinic B. This could be due to presence of enabling health system factors at clinic A not available at clinic B such as well-trained club facilitators. Unexpectedly, patients who had a more recent diagnosis of NCD had better controlled disease compared to patients with an earlier diagnosis. On the same note, women were observed to be more likely to control their NCD compared to men even though this association was not statistically significant (p = 0.072). On multivariate analysis of factors associated with NCD control at 12 months post IC, the association between these variables and NCD control was not statistically significantly at α = 0.05 (Table 4).

**Table 3:** Baseline characteristics associated with control of NCD

Base line characteristic	Controlled NCD N(%)		p value
Age category			
< 40	16 (19.8)		
40–50	38 (46.9)		
> 50	27 (33.3)	0.974	
Sex			
Male	25 (30.9)		
Female	56 (69.1)	0.072	
NCD			
DM only	2 (2.5)		
HTN only	74 (91.4)		
HTN and DM	5 (6.2)	0.430	
BMI			
< 18.5	0 (0)		
18.5–< 25	9 (20.9)		
25–< 30	9 (20.9	0.654	
> 30	25 (58.1)		
WHO stage			
0	25 (30.9)		
1	21 (25.9)		
2	26 (32.1)		
3	3 (3.7)		
4	6 (7.4)	**0.012**	
CD4 count category			
< 350	35 (61.4)		
> 350	22 (38.6)	0.404	
Duration on ART			
0–5	59 (72.8)		
6–10	22 (27.2)		
> 10	0 (0)	0.486	
Duration with HIV			
0–5	37 (58.7)		
6–10	18 (28.6)		
> 10	8 (12.7)	0.537	
Duration with NCD			
0–5	36 (72)		
6–10	13 (26.0)		
> 10	1 (2.0)	**0.004**	
Good adherence			
No	4 (4.94)		
Yes	77 (95.1)	0.204	
Facility			
A	33 (40.7)		
B	48 (59.6)	**0.004**	

Bold p values indicated statitistical significance

**Table 4:** Factors associated with NCD control at 12 months post IC enrolment

Characteristic	Univariate analyses			Multivariate analyses		
	OR^a^	95% CI	p*-*value	aOR^b^	95% CI	p-value
CD4 count at HIV diagnosis (cells/µL)						
< 350	1.00			1.00		
≥ 350	0.67	(0.35, 1.27)	0.217	0.59	(0.29, 1.18)	0.138
Time since NCD diagnosis (years)						
0–5	1.00			1.00		
6–10	0.27	(0.09, 0.83)	**0.022**	0.35	(0.10, 1.16)	0.087
> 10	0.68	(0.12, 3.79)	0.656	1.42	(0.22, 9.25)	0.716
Good adherence (> 80%)						
No	1.00			1.00		
Yes	0.47	(0.19, 1.19)	**0.112**	0.47	(0.16, 1.38)	0.17
Clinic site						
Clinic A	1.00					
Clinic B	1.56	(0.85, 2.87)	**0.150**	1.50	(0.71, 3.16)	0.284
Age group (years)						
< 40	1.00					
40–50	1.14	(0.55, 2.34)	0.729			
> 50						
Sex						
Male	1.00					
Female	1.09	(0.58, 2.03)	0.794			
Comorbidity						
DM	1.00					
DMHTN	0.46	(0.06, 3.36)	0.447			
HTN	1.00	(0.12, 8.42)	1			
BMI category						
< 18.5	1.00					
18.5–24.9	0.47	(0.026, 8.52)	0.610			
25.0–29.9	0.89	(0.05, 15.44)	0.939			
≥ 30	0.54	(0.032, 8.90)	0.666			
WHO stage at HIV diagnosis						
0	1.00					
1	0.78	(0.39, 1.56)	0.483			
2	0.94	(0.48, 1.87)	0.866			
3	2.03	(0.76, 5.41)	0.157			
4						
Time on ART (years)						
0–5	1.00					
6–10	0.96	(0.51, 1.79)	0.896			
> 10	1.97	(0.12, 31.99)	0.634			
Time since HIV diagnosis (years)						
0–5	1.00					
6–10	1.26	(0.69, 2.28)	0.443			
> 10	1.23	(0.51, 2.96)	0.637			

*DM* Diabetes mellitus, *HTN* Hypertension, *DMHTN* Dual diagnosis of hypertension and diabetes mellitus

^a^OR = unadjusted odds ratio

^b^aOR = adjusted odds ratio, p values in bold significant at α = 0.2 level

Bold p values indicated statitistical significance

In univariate logistic regression models, time with NCD, adherence status and IC clinic site at baseline were associated with control of NCD at α = 0.2 level (Table 4). However, on multivariate analysis, the association between these variables and NCD control 12 months post-IC enrolment was not statistically significantly at α = 0.05 (Table 4).

## Discussion

This is the first study to report treatment outcomes among PLHIV diagnosed with NCDs attending integrated NCD/ ART clubs piloted in Cape Town, South Africa. Several studies have reported treatment outcomes in various models of integrated HIV and NCD care. The models of care for HIV/NCD integration have been summarized before into four as: (i) integration of NCD screening and treatment services into established HIV centres [16–18]; (ii) integration of HIV screening and treatment services into established NCD centres; (iii) simultaneous integration of HIV and NCD services at health facilities [19–21] and (iv) integrated HIV and NCD care specifically for multi-morbid patients [22, 23]. However, none of studies has evaluated treatment outcomes among PLHIV with comorbid NCDs attending IC in form of adherence clubs in particular.

This study had several notable findings. Firstly, adherence to group visits (club attendance) was high and sustained at 1-year of attending IC. At 1-year post IC registration, 93.1% of our population was retained in care. While 6.9% of PLHIV were lost to follow up in our study, they may have continued to receive care at an alternative clinic, as is common in this setting.

Secondly, HIV control was sustained at 1-year post IC enrolment with optimal viral suppression near 100%. This finding is reassuring as observed viral suppression, adherence and retention rates are similar to those reported in ordinary MACs in this setting [14, 31, 32]. This demonstrates good progress towards the UNAIDS 90:90:90 targets for HIV epidemic control (at least 90% of PLHIV receiving ART should have suppressed viral loads) [33]. In addition, this finding demonstrates that NCD care can be safely incorporated into HIV care programs without compromising HIV care, thus supporting the notion of leveraging HIV infrastructure for NCD care in the context of the rising NCD epidemic among PLHIV. In addition to increased efficiency in terms of optimizing utilization of resources, the integration of HIV and NCD care has also been reported to be convenient and acceptable to patients [34].

Thirdly, low NCD control rates were found before IC enrolment with an increase in proportion of participants with optimal control at IC enrolment (most likely due to the fact that control of comorbidities was a requirement for eligibility for IC enrolment) but a decline in optimal control at 1-year post IC enrolment was observed. The reason for this decline post IC enrolment is unclear. One possible reason is insufficient exposure to regular NCD-specific health promotion counselling after IC enrolment and thus a lack of support for behaviour modification post IC enrolment. Patients may also have been motivated to adhere to NCD counselling advice prior to IC in order to become eligible for IC enrolment and benefit from the additional conveniences of IC one of which is reduced waiting time at the facility. The absence of sustained NCD health promotion in IC clinics to support maintenance of positive behaviour changes, may have removed the necessary reinforcement required to maintain disease control after enrolment into IC. As NCD control is not simply a product of medication adherence but also requires modification of behavioural factors such as diet, smoking and physical exercise, insufficient support (for example for healthy food security) for modification of such behaviours may have contributed to the lapse in NCD control post IC enrolment [35–37]. Similarly, unmeasured health system factors such as changes to health personnel or other aspects of service delivery in the period post IC enrolment could also have potentially negatively affected NCD control.

Three variables at baseline were associated with control of NCD after 12 months of attending IC. These are: WHO stage at base line, time with NCD and facility. Clinic A was associated with better NCD control compared to clinic B. This could be due to presence of enabling health system factors at clinic A not available at clinic B such as well-trained club facilitators. Contrary to common sense, this study found out that patients who had a more recent diagnosis of NCD controlled their NCD better than the so called “veterans” who had been diagnosed with NCD a little earlier before club enrolment. The idea of “veterans” getting “used to their diagnosis’ may explain this phenomenon. The association of WHO stage with NCD control may be due to the fact that people diagnosed with HIV at earlier stages may have been more physically capable to adopt healthier life -styles such as physical exercise compared to sicker patient presenting in later WHO stages. Therefore, from this finding, people starting clubs in later WHO stages and those presenting with ‘old” NCD at base line may need more support to promote NCD control.

The high proportion of patients with poor NCD, particularly DM, control 12 months before IC enrolment, highlights the burden of poorly controlled NCDs among PLHIV receiving vertical, non-integrated care. This has a negative impact on quality of life for PLHIV as it increases risk of vascular events such as stroke and microvascular events such as renal and ophthalmic disease in addition to ART and HIV complications, thereby exacerbating mortality and morbidity among PLHIV. The higher rates of NCD control at IC enrolment in our study population show the potential of streamlined and intensified care in achieving greater NCD control among PLHIV with MM.

The proportion of participants with optimal BP and glycaemic control 1-year post IC attendance in our study are comparable to those found by Oluwatoyin et al. [23] who described clinical outcomes among PLHIV with comorbid NCD in the USA attending a similar model of integrated care(model iv). In that study, approximately 50% of PLHIV with comorbid DM were reported to have achieved glycaemic control and 47% of PLHIV with HTN were reported to have achieved BP control. Rates of NCD control in our study are also consistent with findings from a study in Uganda that described NCD outcomes among patients upon simultaneous integration of HIV and NCD services at a health facility (model iii) in which a BP control rate of 46% was achieved after attending integrated care for 3 years[21]. Of note, our NCD control rates are considerably higher (by 20% in HTN and 10% in DM) than NCD control rates reported among HIV-negative patients also receiving care at public primary health care facilities in the same Cape Town setting where 33% and 42% of patients with HTN and DM were reported to have optimally controlled BP and diabetes respectively [38]. This may be due to increased access to adherence counselling and retention support that PLHIV received compared to their HIV- negative counterparts as was observed in a Ugandan study by Kwarisiima et al. [21].

Our study had several limitations. Socioeconomic variables known to independently affect HTN and DM control such as income, level of education and behavioural factors (smoking, diet and exercise) [39, 40] were not available from routinely collected patient data and hence were not included in data collection and analysis. As a result, we do not know the impact of these factors on NCD outcomes in our study population. A single blood pressure measurement, recorded in medical folders, was used to assess control 12 months after IC enrolment. This may not sufficiently capture the extent of clinical control for patients with hypertension. We also acknowledge the fact that by 12 months post IC enrolment, almost 7% of patients were lost to follow up(6.9%). In addition, among the patients still in care at 12 months, the proportion that had a measured viral load, blood pressure or HbA1c varied from 66–85% (Fig. 1). This could have resulted in retention bias if patients with worse NCD outcomes were less likely to be lost to follow up or were more likely to have a measured test of disease control recorded. However, evidence from previous studies that have demonstrated that patients lost to follow up are less likely to be adherent to medication [41–43] make this unlikely. Furthermore, a proxy for adherence was used which may not correctly capture true adherence and its impact on NCD control since medication collection may be a necessary but not sufficient factor for medication adherence. We therefore recommend future studies to assess NCD clinical outcomes such as blood pressure at multiple time points and well -designed prospective cohort studies to minimize measurement error and potential bias due to attrition.

Lastly, while univariate logistic regression analyses showed that time with NCD, adherence status and clinic site at IC enrolment were associated with control of NCD at 1-year post IC enrolment, multivariate analysis did not reveal any statistically significant associations. This could be partly due to the small sample size resulting into low statistical power in our multivariate logistic model. However, as we included the entire population of eligible participants without sampling, numbers were ultimately restricted by the low total number of patients to begin with attending IC clinics at the time the study was conducted.

## Conclusion

Our study demonstrated that adults with HIV/NCD multimorbidity can sustain high levels of HIV control following enrolment into integrated NCD and ART care as evidenced by high levels of adherence, viral suppression and minimal loss to follow-up. This evidence supports the move towards integration of NCD care into routine HIV care, leveraging pre-existing HIV infrastructure for NCD care, particularly in high HIV-burden settings undergoing rapid epidemiological transition with a rise in NCD burden and MM. However, we note that NCD control was suboptimal. Therefore, intensified NCD-specific health promoting interventions, upon enrolment into integrated care are needed, particularly for patients with advanced HIV disease or a long NCD history, to sustain NCD control in the long term.

## Supplementary Information

**Additional file 1: Table S1.** Pharmacotherapy for HTN among PLWH with comorbid HTN only compared to pharmacotherapy for HTN among PLWH with comorbid DM and HTN.

## Data Availability

The dataset analysed is available from the corresponding author on reasonable request.

## Abbreviations

HIV: Human immunodeficiency virus
NCD: Non-communicable disease
ART: Ante- retroviral therapy
PLHIV: People living with HIV
MM: Multimorbidity
DM: Diabetes mellitus
HTN: Hypertension
DMHTN: Dual diagnosis of diabetes mellitus and hypertension
IC: Integrated care clubs
SEMSA: Society of Endocrinology, Metabolism and Diabetes of South Africa
HbA1c: Proportion of glycosylated haemoglobin
BP: Blood pressure
SBP: Systolic blood pressure
DBP: Diastolic blood pressure
MACs: Medical adherence clubs
BMI: Body mass index
WHO: World Health Organization
