# Supplementary material for: Treatment outcomes among adults with HIV/non-communicable disease multimorbidity attending integrated care clubs in Cape Town, South Africa
Source: AIDS Res Ther. 2021 Oct 14;18:72. doi: 10.1186/s12981-021-00387-3 (PMC8515722; doi:10.1186/s12981-021-00387-3)
Supplement: Supplementary file 1 — Additional file 1: Table S1. Pharmacotherapy for HTN among PLWH with comorbid HTN only compared to pharmacotherapy for HTN among PLWH with comorbid DM and HTN. [file 12981_2021_387_MOESM1_ESM.pdf]

Supplementary Table 1: Pharmacotherapy for HTN among PLWH with comorbid HTN only compared to pharmacotherapy for HTN among PLWH with comorbid DM and HTN

Name of Anti-hypertensive	HTN only(N,%)	HTN and DM(N,%)	Total(N)	P-value
As initial agent				
Furosemide	2(0.9)	0(0)	2	
Hydrochlorothiazide	175(79.2)	9(52.9)	184	
Enalapril	21(9.5)	3(17.7)	24	
Amlodipine	13(5.9)	3(17.7)	16	
None	10(4.5)	2(11.8)	12	0.112
As additional agent				
Furosemide	1(0.5)	0(0)	1	
Hydrochlorothiazide	15(6.8)	1(6.3)	16	
Enalapril	94(42.5)	10(62.5)	104	
Amlodipine	48(21.7)	2(12.5)	50	
Atenol	4(1.8)	1(6.3)	5	
None	59(26.7)	2(12.5)	61	0.304
